# Solitary Cysticercosis of Parotid Gland Diagnosed on FNAC

**Published:** 2014-04-01

**Authors:** Prashant Goyal, Soumyesh Ghosh, Shelly Sehgal, Deepti Mittal, Sompal Singh

**Affiliations:** Department of Pathology, Swami Dayanand Hospital, Dilshad Garden, Delhi-110095. India

**Dear Sir,**

Cysticercosis is a systemic parasitic disease caused by the larval form of Taenia solium. Cysticercus can be found in any part of the body including the subcutaneous tissues, skeletal muscles, eye, heart, liver, lungs, and peritoneum. Solitary cysticercosis of the parotid gland is rare entity and only few cases have been reported in English literature.[1-3] We report a case of cysticercosis within the parotid gland which was clinically misdiagnosed as parotid gland tumor.

A 13-year-old girl presented with slowly growing painless mass at right preauricular region for the past one year. There was no history of fever, pain, or weight loss. Local examination revealed a rounded 2 cm x2 cm sized, soft-to-firm non-tender mass. The overlying skin showed no signs of inflammation. No cervical lymphadenopathy was noted. Routine blood count including absolute eosinophil count; biochemistry and urine analysis were within normal limits. The clinical diagnosis was that of a parotid gland tumor. FNAC yielded few drops of clear fluid with granular particles. The smears were air dried and stained with May-Grunwald-Giemsa (MGG) stain. On cytology, fragments of bluish fibrillary material with interspersed small nuclei were seen (Fig.1). Fair number of lymphocytes, palisading histiocytes, and degenerated cells in dirty granular background were noted. No eosinophil, granuloma, giant cells or atypical cells were seen. A diagnosis of cysticercosis of parotid gland was made. The patient was advised antihelminthic therapy which resulted in complete resolution of the parotid swelling

**Figure:**
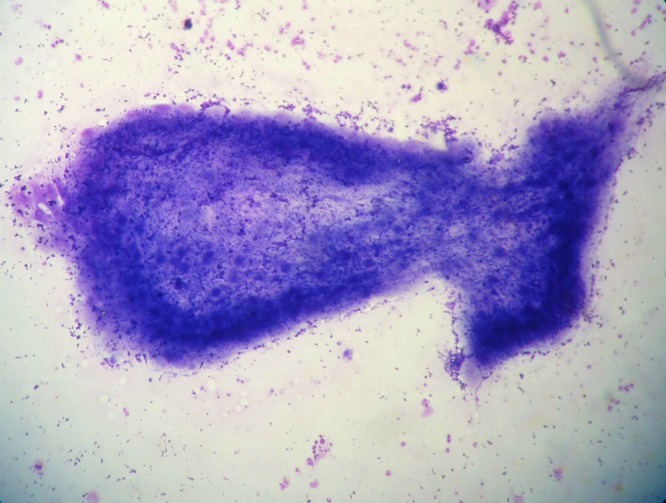
Figure 1: Cytological smear showing bladder wall fragment of cysticercus cellulosae surrounded with inflammatory cells (MGG stain, 40x)

Signs and symptoms of cysticercosis depend on the site of involvement and the stage of evolution. However, the most common presentation is swelling at the affected site. It is commonly misdiagnosed as soft-tissue tumor. In our case, the preoperative diagnosis was a parotid tumor and radiological imaging was in plan but FNAC changed our management plan. In previously reported cases, cysticercosis of parotid presented as mild swelling, or masquerading as a salivary gland neoplasm or with features resembling acute parotitis.[1-5] Sometime, cysticercosis of parotid gland is secondary involvement in case of disseminated cysticercosis.

Definitive diagnosis can be made on FNAC or open biopsy. FNAC is now being increasingly accepted as a reliable, out-patient diagnostic procedure for preoperative diagnosis and may even obviate the need for open biopsy as in our case. In conclusion, cysticercosis should be considered in the differential diagnosis of parotid lump in endemic area with FNAC as the diagnostic tool.

## Footnotes

**Source of Support:** Nil

**Conflict of Interest:** None declared

